# In Vitro Differential Virucidal Efficacy of Alcohol-Based Disinfectants Against Human Norovirus and Its Surrogates

**DOI:** 10.3390/microorganisms13020368

**Published:** 2025-02-08

**Authors:** Eri Hiraishi, Keita Ozaki, Moe Yamakami, Tempei Akasaka, Hirokazu Kimura

**Affiliations:** 1 Department of Research and Development, Niitaka Co., Ltd., Osaka 532-8560, Japan; hiraishi510@niitaka.co.jp (E.H.); ozaki420@niitaka.co.jp (K.O.); yamakami688@niitaka.co.jp (M.Y.); akasaka341@niitaka.co.jp (T.A.); 2 Department of Health Science, Gunma Paz University Graduate School of Health Sciences, Takasaki 370-0006, Japan

**Keywords:** human norovirus, murine norovirus, feline calicivirus, inactivation, alcohol-based disinfectant, hand sanitizer, environmental disinfectant

## Abstract

Human norovirus (HuNoV) is a major causative agent of foodborne illness and causes acute viral gastroenteritis. This study aimed to compare the virucidal efficacies of alcohol-based disinfectants against HuNoV and its surrogates for murine norovirus and feline calicivirus using a cell culture infectivity assay. Additionally, the study evaluated the validity of estimating virucidal efficacy on HuNoV from the results of virucidal efficacy on the surrogate virus. All disinfectants decreased the titer of each virus by >3 log_10_ and >4 log_10_ for an exposure duration of 30 s against murine norovirus and feline calicivirus, respectively. However, acidic alcohol-based disinfectants completely inactivated the HuNoV GII.17 strain for 30 or 60 s, whereas an alkaline alcohol-based disinfectant did not inactivate HuNoV GII.17 for 60 s. This finding indicates that the pH of alcohol disinfectants affects their virucidal effects against HuNoV, and acidity has a higher virucidal efficacy against HuNoV than alkalinity. Disinfectants showing virucidal efficacy against surrogates were not effective against HuNoV. Few studies have used cell culture infectivity assays to test the inactivating effects of hand sanitizers on HuNoV and its surrogates. Our study provides useful information for the development of disinfectants that are effective against HuNoV.

## 1. Introduction

Norovirus belongs to the genus *Norovirus* in the family *Caliciviridae*. It is a nonenveloped virus with an icosahedral capsid and a diameter of 27–40 nm [1]. Noroviruses are classified into 10 groups (GI–GX), of which GI, GII, GIV, GVIII, and GIX infect humans [2]. The norovirus genome is a positive-sense, single-stranded RNA with a length of approximately 7.5 kb [2]. Except for murine norovirus (MNoV), the norovirus genome contains three open reading frames (ORFs), with ORF1 encoding nonstructural proteins as well as ORF2 and ORF3 encoding viral protein 1 (VP1) and VP2, respectively.

Human norovirus (HuNoV) infects humans and causes acute viral gastroenteritis [3]. HuNoV is highly infectious and transmission to humans can occur by only a few dozen viruses that enter through the mouth [4]. HuNoV infection is easily spread through human–object–human or human–human contact transmission [5]. Because there is no effective vaccine or medicine against HuNoV, transmission must be prevented. Alcohol-based disinfectants are widely used in healthcare and food production facilities because of their ease of use due to quick drying, low residue, low corrosion, and low skin irritation. However, alcohol is not effective against some nonenveloped viruses, including HuNoV. Therefore, developing alcohol-based disinfectants that are effective against HuNoV is desirable.

For many years, no artificial cell culture method was established for HuNoV. The following two methods have been mainly used to estimate the inactivation of HuNoV: (i) cell culture infectivity assay using MNoV, feline calicivirus (FCV), and Tulane virus (TV), which are genetically similar to HuNoV and can be cultured in vitro [6,7,8,9,10,11,12,13,14,15,16]; and (ii) detection by gene amplification of HuNoV (reverse transcription-polymerase chain reaction [RT-PCR] assay) [8,9,10,11,17,18,19]. However, both assessment methods have limitations. In cell culture infectivity assays, MNoV, FCV, and TV have been used as HuNoV surrogate viruses. FCV belongs to the same family, *Caliciviridae*, as HuNoV, but it is associated with upper respiratory tract disease in cats [20] and exhibits different physicochemical properties than norovirus [11,21]. However, MNoV causes modest intestinal pathology in wild-type mice [22], and TV causes diarrhea and duodenal inflammation in rhesus macaques [23]. The receptors for MNoV are CD300lf and CD300ld [24]; however, TV recognizes histo-blood group antigens in a manner similar to HuNoV [25]. Thus, HuNoV infection may be more closely related to TV than FCV and MNoV infection. However, the method for evaluating TV inactivation has some difficulties, such as the need to obtain TV samples from positive stool samples or other researchers. In contrast, FCV and MNoV samples are available as products from distribution institutions, such as the American Type Culture Collection (ATCC), making evaluation easier than that with TV. The guidelines of the Centers for Disease Control and Prevention (CDCs) mention FCV and MNoV as examples of HuNoV surrogate viruses [26]. Furthermore, it is recommended that the virucidal efficacy of disinfectants should be evaluated using multiple surrogate viruses rather than a single surrogate virus [26]. Therefore, even after the HuNoV culture system was established using human intestinal enteroids [27], virucidal efficacy has been evaluated using the surrogate viruses FCV and MNoV [28,29]. This estimation is based on the hypothesis that MNoV, FCV, and HuNoV have similar susceptibilities to disinfectants. However, these viruses have different susceptibilities to chemical substances, such as alcohol, acid, and alkali [11,21]. Therefore, there might be differences in the virucidal efficacy of alcohol-based disinfectants between surrogate viruses and HuNoV. It is necessary to verify whether disinfectants effective against both FCV and MNoV are effective against HuNoV. In the RT-PCR assay, viral inactivation is evaluated based on the amplification of the HuNoV genome before and after disinfectant treatment. This method detects only decreases in viral genes due to disinfectant treatment and does not determine whether the virus is infectious to the cells. For poliovirus 1 or FCV, even if viral genes are detected by RT-PCR, they do not indicate the presence of an infectious virus [30]. Therefore, it is possible that the RT-PCR assay does not accurately evaluate viral inactivation.

Since 2016, artificial culture methods have been established for HuNoV [27,31], making it possible to evaluate HuNoV inactivation using cell culture infectivity assays. Several studies have evaluated the virucidal efficacy of disinfectants against HuNoV, including alcohol [32,33,34,35]. However, to our knowledge, no studies have evaluated the correlation between virucidal efficacy against HuNoV and its surrogates using a cell culture infectivity assay. By comparing the susceptibility of these viruses to disinfectants, we provide insights for estimating the virucidal efficacy against HuNoV from the inactivation efficacy of surrogates. In this study, we compared the efficacy of four commercially available alcohol-based disinfectants and 70% ethanol against HuNoV, MNoV, and FCV using a cell culture infectivity assay.

## 2. Materials and Methods

### 2.1. Cells

RAW 264.7 cells (TIB-71, ATCC, Manassas, VA, USA) were cultured at 37 °C under a 5% CO_2_ atmosphere in Dulbecco’s modified Eagle’s medium (DMEM, Merck, Darmstadt, Germany) containing 10% heat-inactivated fetal bovine serum (FBS, Biowest, Nuaillé, France), 100 U/L penicillin, and 100 mg/L streptomycin (Fujifilm Wako, Osaka, Japan). Crandell–Rees feline kidney (CRFK) cells (CCL-94, ATCC) were cultured at 37 °C under a 5% CO_2_ atmosphere in DMEM containing 10% heat-inactivated FBS and 5 mg/L gentamicin sulfate (Fujifilm Wako). Human induced pluripotent stem cell-derived small intestinal epithelial-like cells (F-hiSIEC, Fujifilm, Tokyo, Japan) were cultured according to the manufacturer’s instructions.

### 2.2. Viruses

MNoV strain S7 (a gift from Dr. Yukinobu Tohya, Nihon University) was propagated in RAW 264.7 cells using DMEM containing 10% heat-inactivated FBS, 100 U/L penicillin, and 100 mg/L streptomycin. FCV strain F9 (VR-782, ATCC) was propagated in CRFK cells using a modified Eagle’s minimum essential medium (Opti-MEM, Gibco, Thermo Scientific, Waltham, MA, USA). Confluent monolayers of RAW 264.7 or CRFK cells in T-150 cm^2^ cell culture flasks were infected with MNoV or FCV at a multiplicity of infection of 0.001 to prepare virus stocks and incubated at 37 °C under 5% CO_2_ atmosphere until a complete cytopathic effect was apparent. The virus suspension was harvested from the cell supernatant followed by centrifugation (CF16RN, Himac, Ibaraki, Japan) at 3500× *g* for 20 min at 4 °C. The virus was stored in aliquots at −80 °C until use. The titers of MNoV and FCV stocks were 5.9–7.3 log_10_ median tissue culture infectious dose (TCID_50_)/50 µL and 5.9–6.7 log_10_ TCID_50_/100 µL, respectively.

GII.17 HuNoV was collected from the stool of a patient positive for HuNoV. To prepare 10% stool suspensions, HuNoV-positive stool was suspended in phosphate-buffered saline (PBS, Fujifilm Wako) followed by mixing in a bioshaker (BR-23FP, TAITEC, Saitama, Japan) at 300 rpm for 20 min. The suspension was centrifuged at 10,000× *g* for 5 min at 4 °C. The supernatant was passed serially through 5-, 0.8-, 0.45-, and 0.22-µm filters (Merck). The filtered 10% stool suspension was aliquoted and frozen at −80 °C until use. A HuNoV suspension was prepared by diluting tenfold to 3.6 × 10^4^ genome equivalents (GEs)/µL with PBS immediately before use.

### 2.3. Disinfectant Solutions

Four alcohol-based disinfectant products (A–D) manufactured by Niitaka Co., Ltd. (Osaka, Japan) were obtained (Table 1). Then, 70 wt% ethanol was prepared by diluting ≥ 99.5% ethanol (Sigma-Aldrich, St. Louis, MO, USA) with distilled water.

### 2.4. Assessment of Disinfectant Solutions Against MNoV and FCV

A mixture of 10% beef extract (Nacalai Tesque, Kyoto, Japan) and MNoV or FCV suspension was prepared at a ratio of 1:1. Five microliters of MNoV or FCV suspension containing 5% beef extract was mixed with 45 µL of each disinfectant solution or medium (DMEM for MNoV and Opti-MEM for FCV) and incubated at room temperature for 30 or 60 s. Ten microliters of each suspension were quickly added to 1990 µL of medium (DMEM containing 10% FBS for MNoV and Opti-MEM for FCV) to neutralize their virucidal activity. For MNoV, the diluted sample was added to RAW264.7 cells (initial cell seeding concentration: 2.5 × 10^4^ cells/well) and cultured for 3 h after seeding on a 96-well microtiter plate. For FCV, the diluted sample was added to confluent monolayers of CRFK cells (initial cell seeding concentration: 8.0 × 10^3^ cells/well) cultured at 37 °C under 5% CO_2_ atmosphere for 3 or 4 days after seeding on a 96-well microtiter plate. The cells were cultured at 37 °C under an atmosphere containing 5% CO_2_ for 4 days. A cytopathic effect was observed, and TCID_50_ was calculated from the cytopathic effect using the Behrens–Kärber method [36].

### 2.5. Assessment of Disinfectant Solutions Against HuNoV

Briefly, 10 µL of HuNoV suspension containing 3.6 × 10^4^ GEs/µL was mixed with 90 µL of each disinfectant solution and incubated at room temperature for 30 or 60 s. Each virus mixture was quickly mixed with 1000 µL of Advanced DMEM/F-12 (Gibco, Thermo Scientific) to dilute the disinfectant 11-fold. To remove the disinfectant components and concentrate virus, the dilution was ultracentrifuged using Optima MAX-TL with a type TLA-55 rotor (Beckman Coulter, Brea, CA, USA) at 55,000 rpm for 90 min at 4 °C. The supernatant was removed, and the pellet was resuspended in 100 µL of culture medium for F-hiSIECs containing 500 µM sodium glycochenodeoxycholate (Sigma-Aldrich). As a result, 1100 µL of a solution containing disinfectant-treated virus and the medium was concentrated to 100 µL—an 11-fold concentration—matching the original volume used when the virus suspension and disinfectant were mixed. One-half of the resuspended mixture (50 µL) was transferred to two confluent monolayers of F-hiSIECs seeded on a 96-well microtiter plate, followed by 3 h of incubation at 37 °C under 5% CO_2_ atmosphere. The inoculum was removed, and the F-hiSIEC monolayers were washed twice with DMEM/F-12 (Gibco, Thermo Scientific). One hundred microliters of culture medium for F-hiSIECs containing 500 µM sodium glycochenodeoxycholate was added to the monolayers. Of the two identical plates, the monolayers and supernatants on one plate were harvested with 300 µL of TRIsure (Bioline, London, UK) at 3 h post-infection (hpi). Cells and supernatants on the other plate were incubated at 37 °C under a 5% CO_2_ atmosphere for 69 h and harvested with TRIsure at 72 hpi. An untreated control was tested in the same manner, replacing the disinfectant solution with PBS.

### 2.6. Quantification of the HuNoV Genome

HuNoV RNA was extracted from F-hiSIEC monolayers and the supernatants at 3 and 72 hpi using Direct-zol RNA MiniPrep Kit (Zymo Research, Irvine, CA, USA) following the manufacturer’s instructions. RNA was eluted in 50 μL of distilled water.

HuNoV RNA GEs of 3 and 72 hpi were determined by reverse transcription-quantitative PCR (RT-qPCR) using CFX Opus Deepwell Real-Time PCR System (Bio-Rad, Hercules, CA, USA) in a 20 µL reaction volume targeting the conserved ORF1–ORF2 junction of GII HuNoV. The primer pair COG2F/COG2R and the probe RING2AL-TP were used to detect HuNoV RNA using Luna Universal Probe One-Step RT-qPCR Kit (New England Biolabs, Ipswich, MA, USA) according to the manufacturer’s instructions [37]. Five microliters of RNA extract solution were used to detect HuNoV RNA in each sample. A standard curve generated using a plasmid containing HuNoV genome sequences was used to quantify viral GEs in the RNA samples. The limit of detection in RT-qPCR analysis was defined as 5 GEs per reaction (2.4 log_10_ GEs/well) based on a standard curve.

### 2.7. Statistical Analysis

Experimental data for the surrogate virus and HuNoV were collected from three and two independent experiments, respectively. Data were indicated as mean ± standard deviation. Data were compared using Welch’s *t*-test and Kruskal–Wallis test followed by Steel’s test using Microsoft Excel version 2406 and Statcel 4 software (OMS Publishing, Saitama, Japan), respectively. A *p*-value < 0.05 was considered statistically significant.

### 2.8. Ethical Statement

The studies involving human stool samples were approved by the bioethics committee of Niitaka Co., Ltd. (approval number 22-02). Patients provided written informed consent to participate in this study.

## 3. Results

### 3.1. Virucidal Activities of Disinfectant Solutions Against MNoV and FCV

We assessed the efficacy of 70 wt% ethanol and four disinfectant products against HuNoV surrogates, MNoV and FCV. To simulate real-world conditions, an assay with an exposure duration of 30 or 60 s was performed using virus mixtures combined equally with 10% beef extract as organic matter. No cytotoxicity was observed under any condition when the cells were treated with a disinfectant solution diluted 200-fold with medium, and the disinfectants diluted with the medium neutralized their virucidal activity (Table S1). First, we assessed the virucidal efficacy against MNoV. The MNoV infectivity titer significantly decreased after 30 s of treatment with 70 wt% ethanol compared with the untreated medium control (6.8 ± 0.1 log_10_ TCID_50_/50 µL with untreated versus < 1.9 ± 0.3 log_10_ TCID_50_/50 µL with 70 wt% ethanol, *p* < 0.01, Table 2). Further, 70 wt% ethanol decreased the MNoV titer by >4.9 ± 0.4 log_10_. When MNoV was treated with products A–D for 30 s, the titer was significantly decreased compared with the untreated medium control (5.9 ± 0.2 log_10_ TCID_50_/50 µL with untreated versus < 1.8 ± 0.0 log_10_ TCID_50_/50 µL with product A, *p* < 0.01; 6.2 ± 0.2 log_10_ TCID_50_/50 µL with untreated versus 2.9 ± 0.3 log_10_ TCID_50_/50 µL with product B, *p* < 0.01; 6.1 ± 0.2 log_10_ TCID_50_/50 µL with untreated versus < 1.8 ± 0.1 log_10_ TCID_50_/50 µL with product C, *p* < 0.01; and 6.5 ± 0.4 log_10_ TCID_50_/50 µL with untreated versus < 1.8 ± 0.0 log_10_ TCID_50_/50 µL with product D, *p* < 0.01, Table 2). Consequently, the titer reductions for MNoV were >4.9 ± 0.4 log_10_, >4.1 ± 0.2 log_10_, 3.3 ± 0.4 log_10_, >4.3 ± 0.1 log_10_, and >4.7 ± 0.4 log_10_ for treatments with 70 wt% ethanol, product A, product B, product C, and product D, respectively (Table 2). When the exposure duration of product B treatment was extended to 60 s, the reduction in MNoV titer was increased to >4.4 ± 0.2 log_10_ (Table 2).

Next, we assessed the virucidal efficacy against FCV. There was no significant difference in the infectivity titer of 70 wt% ethanol-treated FCV compared with that of the untreated medium control (5.9 ± 0.2 log_10_ TCID_50_/100 µL with untreated versus 6.0 ± 0.3 log_10_ TCID_50_/100 µL with 70 wt% ethanol, Table 3). Further, 70 wt% ethanol did not decrease the FCV titer. In contrast, when FCV was treated with products A–D for 30 s, the titer was significantly decreased compared with that of the untreated control (6.3 ± 0.3 log_10_ TCID_50_/100 µL with untreated versus < 1.8 ± 0.1 log_10_ TCID_50_/100 µL with product A, *p* < 0.01; 6.2 ± 0.3 log_10_ TCID_50_/100 µL with untreated versus < 1.8 ± 0.0 log_10_ TCID_50_/100 µL with product B, *p* < 0.01; 6.3 ± 0.1 log_10_ TCID_50_/100 µL with untreated versus < 1.9 ± 0.1 log_10_ TCID_50_/100 µL with product C, *p* < 0.01; and 6.7 ± 0.2 log_10_ TCID_50_/100 µL with untreated versus < 1.8 ± 0.1 log_10_ TCID_50_/100 µL with product D, *p* < 0.01, Table 3). As a result, the FCV titer reductions were >4.5 ± 0.1 log_10_, >4.4 ± 0.3 log_10_, >4.4 ± 0.1 log_10_, and >4.9 ± 0.1 log_10_ for product A, product B, product C, and product D treatment, respectively (Table 3). Therefore, under the condition of organic matter addition, the four products exhibited high virucidal efficacy against MNoV and FCV. In addition, 70 wt% ethanol effectively inactivated MNoV but did not inactivate FCV.

### 3.2. Virucidal Activities of Disinfectant Solutions Against GII.17 HuNoV

**Figure 1:**
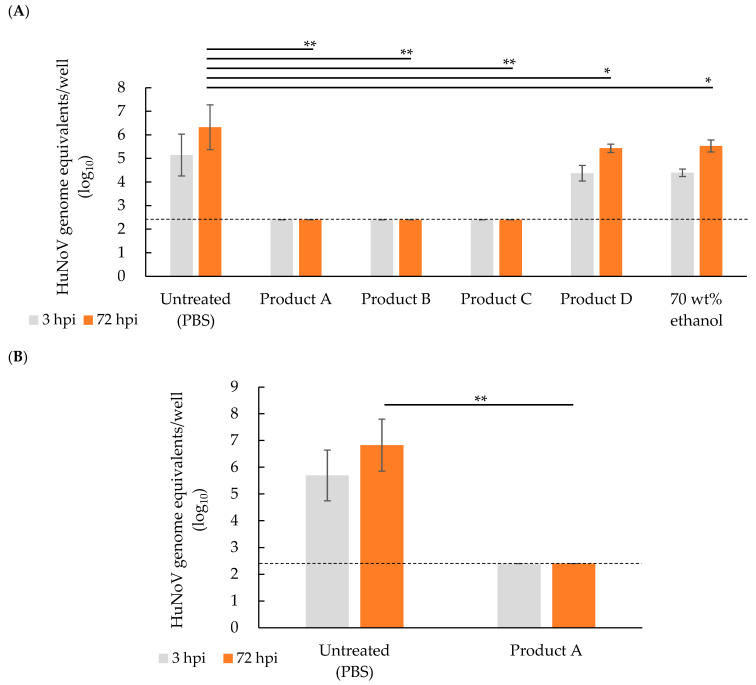
GII.17 HuNoV inactivation by disinfectants containing ethanol. We tested the exposure duration of disinfectant solutions against HuNoV at 60 s (**A**) or 30 s (**B**). HuNoV inactivation assays were performed as described in Materials and Methods. Viral RNA was extracted from both cells and supernatants at 3 and 72 hpi. Viral RNA was quantified using reverse transcriptase quantitative PCR (RT-qPCR). Values on the vertical axis indicate the amount of HuNoV RNA at 3 and 72 hpi. Labels on the horizontal axis indicate the disinfectant solutions used in the HuNoV inactivation experiments. Results are presented as mean ± standard deviation. Each experiment was performed two times. The compiled data represent the RNA mean of three wells. Dashed lines indicate detection limits for the assay. Data were compared using the Kruskal–Wallis test, followed by Steel’s test. Asterisks indicate significant differences between the untreated and each disinfectant-treated group at 72 hpi (* *p* < 0.05 and ** *p* < 0.01).

The efficacy of four disinfectant products and 70 wt% ethanol against GII.17 HuNoV was evaluated at exposure durations of 30 and 60 s. GII.17 HuNoV genomic RNA (at least 1.8 × 10^3^ GEs/well) was required to establish HuNoV infection in F-hiSIECs (Figure S1). HuNoV inactivation tests were performed using HuNoV suspensions containing 1.8 × 10^5^ GEs/well; thus, a maximum of 2 log_10_ reductions in HuNoV GEs was detectable. When disinfectant-treated HuNoV was added to the cells, cytotoxicity was not observed under any condition because of the separation of disinfectant components from HuNoV and each disinfectant mixture by ultracentrifugation. In a test to confirm the neutralizing efficiency of virucidal activity, the amount of HuNoV RNA at 72 hpi for products A–D and 70 wt% ethanol treatment increased between 7- and 28-fold compared with those at 3 hpi (Figure S2). The increase in the amounts of HuNoV RNA was comparable to the 13-fold increase in the PBS control (Figure S2). Thus, it was confirmed that the components contained in the disinfectants did not affect viral proliferation, indicating that the virucidal efficacy of the disinfectants was properly neutralized. We quantified the amount of genomic RNA detected at 72 hpi after treatment with products A–C (acidic disinfectants) for 60 s. After treatment, the amounts were significantly reduced compared with that of the PBS control (6.3 ± 0.9 log_10_ GEs with untreated versus < 2.4 ± 0.0 log_10_ GEs with product A, product B, and product C, *p* < 0.01; Figure 1A) and were below the detection limit. Therefore, we obtained complete inactivation results against HuNoV with products A–C in the culture system. The RNA amount at 72 hpi treated with product D (alkaline disinfectant) or 70 wt% ethanol for 60 s was significantly reduced compared with treatment with the PBS control at 72 hpi (6.3 ± 0.9 log_10_ GEs with untreated versus 5.4 ± 0.2 log_10_ GEs with product D, *p* < 0.05 and 6.3 ± 0.9 log_10_ GEs with untreated versus 5.5 ± 0.3 log_10_ GEs with 70 wt% ethanol, *p* < 0.05, Figure 1A). However, the amount of HuNoV RNA at 72 hpi proliferated 10- and 15-fold compared with those at 3 hpi following treatment with product D and 70 wt% ethanol, respectively, indicating that HuNoV was not inactivated (Figure 1A).

Because product A has been used as a hand sanitizer, it was considered more desirable to inactivate HuNoV for a short duration. Therefore, the efficiency of product A in inactivating HuNoV for a shorter exposure duration (30 s) was evaluated. The amount of HuNoV RNA at 72 hpi for product A was below the detection limit at an exposure duration of 30 s and significantly reduced compared with the PBS control (6.8 ± 1.0 log_10_ GEs with untreated versus < 2.4 ± 0.0 log_10_ GEs with product A, *p* < 0.01, Figure 1B). Therefore, product A completely inactivated HuNoV at only 30 s (Figure 1B). These data showed that the virucidal efficacy against HuNoV differed depending on the pH or ethanol concentration of the alcohol disinfectants, indicating that acidic alcohol disinfectants are more effective than alkaline ones.

## 4. Discussion

This study compared the virucidal efficacy of alcohol-based disinfectant products against HuNoV and two surrogates using a cell culture infectivity assay and evaluated the validity of estimating the virucidal efficacy of HuNoV from the virucidal efficacy of the surrogate virus. The following are the findings: (i) all products containing at least 57.2 wt% ethanol and acid or alkaline agent were highly efficient in inactivating MNoV and FCV in the presence of organic matter for an exposure duration of 30 s. All products reduced virus titers by >3 log_10_ against MNoV and >4 log_10_ against FCV (Table 2 and Table 3); (ii) products A–C, containing at least 57.2 wt% ethanol and acid agents, inactivated HuNoV below the detection limit with an exposure duration of 30 or 60 s (*p* < 0.01, Figure 1), whereas product D, which contained 57.2 wt% ethanol and an alkaline agent, showed no virucidal efficacy against HuNoV at an exposure duration of 60 s (Figure 1). We hypothesized that the pH of the alcohol disinfectant affected its effectiveness against HuNoV, and the acidic alcohol disinfectant was more effective for HuNoV inactivation than the alkaline alcohol disinfectant. These results indicate that the alcohol-based disinfectants used in this study were effective against surrogate viruses but not always against HuNoV.

Ethanol solution has high virucidal efficacy against enveloped viruses [38]. However, its virucidal efficacy against nonenveloped viruses varies according to the type of virus [38]. FCV, a nonenveloped virus, is relatively resistant to ethanol, regardless of concentration, but MNoV showed high sensitivity to ethanol [11]. A study evaluating the sensitivity of surrogate viruses to pH showed that FCV is more likely to be inactivated at ≤pH 3 and ≥10 and that MNoV is less sensitive to pH [21]. FCVs are internalized by cells during infection, and uncoating occurs because of endosomal acidification [39]. Therefore, FCV capsid protein may be particularly unstable and easily inactivated under acidic conditions. The results of this study are similar to those of previous studies. Products A, B, and C contained phosphoric acid, citric acid, and tartaric acid, respectively, and the pH of each product was 3. In contrast, product D contained sodium bicarbonate and had a pH of 9. The four products showed high inactivating effects against both surrogate viruses because they contained high concentrations of ethanol (>57.22 wt%) with a pH of 3.2 or 9.2 (Table 1). FCV may have been inactivated by the combined effects of ethanol and the acidic or alkaline components in the disinfectant, and MNoV was inactivated by the high concentration of ethanol (>57.2 wt%) in the disinfectant.

Several reports have examined the inactivating effect of ethanol against HuNoV. Costantini et al. reported that 70% ethanol could not inactivate five HuNoV GII.4 strains [32]. Sato et al. reported that 70% ethanol was effective in inactivating GII.4 but not GII.3, GII.6, GII.17, or GI.7 [33]. Susceptibility to ethanol may differ depending on the genotype of HuNoV. The strain GII.17 might be more resistant to ethanol than GII.4, which is prevalent worldwide. Similarly, in this study, GII.17 was not inactivated by 70% ethanol (Figure 1A).

Several studies have evaluated the correlation between HuNoV inactivation and pH. Acidic alcohol solutions (pH ~3.1) completely inactivated GII.4 and GII.17 for 30 s of exposure, whereas alkaline alcohol solutions (pH ~11) completely inactivated GII.4 but not GII.17 [33]. The results of this study were similar to those of previous reports. Products A–C, the acidic disinfectants (pH 3.2), completely inactivated GII.17 strains for 30 or 60 s of exposure, whereas product D, the alkaline disinfectant (pH 9.1), was unable to inactivate GII.17 for 60 s of exposure (Figure 1). Abou-Hamand et al. investigated the correlation between HuNoV morphology and pH [40]. The morphology of GII.17 virus-like particle (VLP) changed in a pH-dependent manner. In an acidic solution at pH 2, VLP disassembled completely. At pH 4, VLP aggregated, but in an alkaline solution at pH 9.6, VLP was structurally intact [40]. Based on these findings, the capsid protein surface of GII.17 strains may be susceptible to acidic solutions; thus, GII.17 strains may be easily inactivated. However, because the GII.17 capsid protein was stable in relatively weak alkaline solutions, we hypothesized that the relatively weak alkaline state did not affect viral inactivation. Because GII.17 HuNoV may be inactivated in alkaline solutions, further verification of virucidal efficacy in moderate-to-strong alkaline solutions is needed.

HuNoV may differ in susceptibility to disinfectants depending on genotype [32,33]. Our study showed the effect of disinfectants on only HuNoV GII.17. HuNoV GII.4 caused a worldwide pandemic, and other genotypes caused sporadic epidemics. Although this study demonstrates the importance of evaluation using HuNoV itself rather than surrogates, we recognize that future inactivation studies using various HuNoV genotypes should be conducted to accumulate data to validate the efficacy of disinfectants.

CDC guidelines indicated that disinfectants effective against both MNoV and FCV surrogate viruses, rather than just one, are likely to be effective against HuNoV [26]. Although there are some similarities in the inactivation characteristics of alcohol-based disinfectants against HuNoV and its surrogates, this study showed that effective products against both surrogates were not necessarily effective against HuNoV. A method capable of culturing HuNoV in vitro was developed in 2016 [27] but has not been widely adopted because of the high cost of introducing the culture system and the ethical hurdles in using fecal samples from patients for study. Therefore, the virucidal efficacy against HuNoV has been evaluated using surrogates. The use of HuNoV rather than surrogates is important when evaluating the efficiency of disinfectants in inactivating HuNoV. This information may be useful for developing effective HuNoV disinfectants. However, the currently established in vitro HuNoV culture system cannot quantitatively evaluate viral infection titers because plaque assays are not applicable. Although there is a study in which the amount of HuNoV RNA measured by RT-PCR was applied to the TCID_50_ method, to our knowledge, the evaluation of HuNoV infectivity using the TCID_50_ method is not common [41]. Therefore, it is difficult to evaluate the virucidal efficacy of disinfectants and other agents against HuNoV based on the decreased level of virus infectivity titer. Methods must be developed for HuNoV to quantify viral infective titers in the same way as that for other viruses, such as FCV and MNoV, to accurately evaluate the virucidal efficacy of disinfectants.

## Figures and Tables

**Table 1:** Alcohol-based disinfectants.

Product Name	Active Ingredient		pH	Disinfectant Uses
	Main Component	Concentration		
A	Ethanol Phosphoric acid	72.6 wt% 0.8 wt%	3.2	Hand sanitizer
B	Ethanol Citric acid	57.2 wt% 1.9 wt%	3.2	Environmental disinfectant
C	Ethanol Tartaric acid	67.9 wt% 0.8 wt%	3.2	Environmental disinfectant
D	Ethanol Sodium hydrogen carbonate	57.2 wt% 0.5 wt%	9.1	Environmental disinfectant

**Table 2:** Virucidal activities of disinfectant solutions against MNoV.

Disinfectant Solutions	Exposure Duration [s]	Viral Infectivity Titer [log_10_ TCID_50_/50 µL] ^(a)^		Decrease in Viral Titer [log_10_]
		Untreated	Disinfectant Treatment ^(b)^	
70 wt% ethanol	30	6.8 (0.1)	1.9 ** (0.3)	>4.9 (0.4)
Product A	30	5.9 (0.2)	1.8 ** (0.0)	>4.1 (0.2)
Product B	30	6.2 (0.2)	2.9 ** (0.3)	3.3 (0.4)
	60	6.2 (0.2)	1.8 ** (0.0)	>4.4 (0.2)
Product C	30	6.1 (0.2)	1.8 ** (0.1)	>4.3 (0.1)
Product D	30	6.5 (0.4)	1.8 ** (0.0)	>4.7 (0.4)

^(a)^ Data are shown as mean, and standard deviations are shown in parentheses. ^(b)^ Asterisks indicate significant differences between the untreated and each disinfectant-treated group (** *p* < 0.01). Statistical analysis was conducted using Welch’s *t*-test in Microsoft Excel version 2406.

**Table 3:** Virucidal activities of disinfectant solutions against FCV.

Disinfectant Solutions	Exposure Duration [s]	Viral Infectivity Titer [log_10_ TCID_50_/100 µL] ^(a)^		Decrease in Viral Titer [log_10_]
		Untreated	Disinfectant Treatment ^(b)^	
70 wt% ethanol	30	5.9 (0.2)	6.0 (0.3)	−0.1 (0.3)
Product A	30	6.3 (0.3)	1.8 ** (0.1)	>4.5 (0.1)
Product B	30	6.2 (0.3)	1.8 ** (0.0)	>4.4 (0.3)
Product C	30	6.3 (0.1)	1.9 ** (0.1)	>4.4 (0.1)
Product D	30	6.7 (0.2)	1.8 ** (0.1)	>4.9 (0.1)

^(a)^ Data are presented as mean, and standard deviations are shown in parentheses. ^(b)^ Asterisks indicate significant differences between the untreated and each disinfectant-treated group (** *p* < 0.01). Statistical analysis was conducted using Welch’s *t*-test in Microsoft Excel version 2406.

## Data Availability

The raw data supporting the conclusions of this article will be made available by the authors upon request.

## Supplementary Materials

The following supporting information can be downloaded at https://www.mdpi.com/article/10.3390/microorganisms13020368/s1. More details can be found in the following: 1. Evaluation of neutralizing effect of 70 wt% ethanol and alcohol-based disinfectant products on murine norovirus (MNoV) and feline calicivirus (FCV); Table S1: Evaluation of the neutralizing efficiency of 70 wt% ethanol and disinfectant products against MNoV and FCV; Figure S1: Correlation between the amount of human norovirus (HuNoV) RNA in the HuNoV infection experiments and HuNoV RNA proliferation in monolayer intestinal epithelial cells; Figure S2: Evaluation of the neutralizing efficiency of virucidal activity of products and 70 wt% ethanol against GII.17 HuNoV.

## References

[1] Green K.Y., Knipe D.M., Howley P.M. (2013). Caliciviridae: The Noroviruses. Fields Virology.

[2] Chhabra P., De Graaf M., Parra G.I., Chan M.C.-W., Green K., Martella V., Wang Q., White P.A., Katayama K., Vennema H. (2019). Updated Classification of Norovirus Genogroups and Genotypes. J. Gen. Virol..

[3] World Health Organization (2015). WHO Estimates of the Global Burden of Foodborne Diseases: Foodborne Disease Burden Epidemiology Reference Group 2007–2015.

[4] Caul E.O. (1994). Small round structured viruses: Airborne transmission and hospital control. Lancet.

[5] Lopman B., Gastañaduy P., Park G.W., Hall A.J., Parashar U.D., Vinjé J. (2012). Environmental Transmission of Norovirus Gastroenteritis. Curr. Opin. Virol..

[6] Doultree J.C., Druce J.D., Birch C.J., Bowden D.S., Marshall J.A. (1999). Inactivation of Feline Calicivirus, a Norwalk Virus Surrogate. J. Hosp. Infect..

[7] Duizer E., Bijkerk P., Rockx B., de Groot A., Twisk F., Koopmans M. (2004). Inactivation of Caliciviruses. Appl. Environ. Microbiol..

[8] Park G.W., Boston D.M., Kase J.A., Sampson M.N., Sobsey M.D. (2007). Evaluation of Liquid- and Fog-Based Application of Sterilox Hypochlorous Acid Solution for Surface Inactivation of Human Norovirus. Appl. Environ. Microbiol..

[9] Hewitt J., Rivera-Aban M., Greening G.E. (2009). Evaluation of Murine Norovirus as a Surrogate for Human Norovirus and Hepatitis A Virus in Heat Inactivation Studies. J. Appl. Microbiol..

[10] Girard M., Ngazoa S., Mattison K., Jean J. (2010). Attachment of Noroviruses to Stainless Steel and Their Inactivation, Using Household Disinfectants. J. Food Prot..

[11] Tung G., Macinga D., Arbogast J., Jaykus L.-A. (2013). Efficacy of Commonly Used Disinfectants for Inactivation of Human Noroviruses and Their Surrogates. J. Food Prot..

[12] Li X., Ye M., Neetoo H., Golovan S., Chen H. (2013). Pressure Inactivation of Tulane Virus, a Candidate Surrogate for Human Norovirus and Its Potential Application in Food Industry. Int. J. Food Microbiol..

[13] Arthur S.E., Gibson K.E. (2015). Physicochemical Stability Profile of Tulane Virus: A Human Norovirus Surrogate. J. Appl. Microbiol..

[14] Akasaka T., Shimizu-Onda Y., Hayakawa S., Ushijima H. (2016). The Virucidal Effects against Murine Norovirus and Feline Calicivirus F4 as Surrogates for Human Norovirus by the Different Additive Concentrations of Ethanol-Based Sanitizers. J. Infect. Chemother..

[15] Randazzo W., Costantini V., Morantz E.K., Vinjé J. (2020). Human Intestinal Enteroids to Evaluate Human Norovirus GII.4 Inactivation by Aged-Green Tea. Front. Microbiol..

[16] Huang J., Park G.W., Jones R.M., Fraser A.M., Vinjé J., Jiang X. (2022). Efficacy of EPA-Registered Disinfectants against Two Human Norovirus Surrogates and Clostridioides Difficile Endospores. J. Appl. Microbiol..

[17] Topping J.R., Schnerr H., Haines J., Scott M., Carter M.J., Willcocks M.M., Bellamy K., Brown D.W., Gray J.J., Gallimore C.I. (2009). Temperature Inactivation of Feline Calicivirus Vaccine Strain FCV F-9 in Comparison with Human Noroviruses Using an RNA Exposure Assay and Reverse Transcribed Quantitative Real-Time Polymerase Chain Reaction—A Novel Method for Predicting Virus Infectivity. J. Virol. Methods.

[18] Kamimoto M., Nakai Y., Tsuji T., Shimamoto T., Shimamoto T. (2014). Antiviral Effects of Persimmon Extract on Human Norovirus and Its Surrogate, Bacteriophage MS2. J. Food Sci..

[19] Takahashi H., Nakazawa M., Ohshima C., Sato M., Tsuchiya T., Takeuchi A., Kunou M., Kuda T., Kimura B. (2015). Heat-Denatured Lysozyme Inactivates Murine Norovirus as a Surrogate Human Norovirus. Sci. Rep..

[20] Bennett M., McArdle F., Glenn M.A., Turner P.C., Gaskell R.M., Dawson S., Radford A.D., Williams R.A. (1998). Quasispecies Evolution of a Hypervariable Region of the Feline Calicivirus Capsid Gene in Cell Culture and in Persistently Infected Cats. J. Gen. Virol..

[21] Cannon J.L., Papafragkou E., Park G.W., Osborne J., Jaykus L.-A., Vinjé J. (2006). Surrogates for the Study of Norovirus Stability and Inactivation in the Environment: A Comparison of Murine Norovirus and Feline Calicivirus. J. Food Prot..

[22] Kahan S.M., Liu G., Reinhard M.K., Hsu C.C., Livingston R.S., Karst S.M. (2011). Comparative Murine Norovirus Studies Reveal a Lack of Correlation between Intestinal Virus Titers and Enteric Pathology. Virology.

[23] Sestak K., Feely S., Fey B., Dufour J., Hargitt E., Alvarez X., Pahar B., Gregoricus N., Vinjé J., Farkas T. (2012). Experimental Inoculation of Juvenile Rhesus Macaques with Primate Enteric Caliciviruses. PLoS ONE.

[24] Haga K., Fujimoto A., Takai-Todaka R., Miki M., Doan Y.H., Murakami K., Yokoyama M., Murata K., Nakanishi A., Katayama K. (2016). Functional Receptor Molecules CD300lf and CD300ld within the CD300 Family Enable Murine Noroviruses to Infect Cells. Proc. Natl. Acad. Sci. USA.

[25] Zhang D., Huang P., Zou L., Lowary T.L., Tan M., Jiang X. (2015). Tulane Virus Recognizes the A Type 3 and B Histo-Blood Group Antigens. J. Virol..

[26] Hall A.J., Vinjé J., Lopman B., Park G.W., Yen C., Gregoricus N., Parashar U. (2011). Updated norovirus outbreak management and disease prevention guidelines. MMWR Recomm. Rep..

[27] Ettayebi K., Crawford S.E., Murakami K., Broughman J.R., Karandikar U., Tenge V.R., Neill F.H., Blutt S.E., Zeng X.-L., Qu L. (2016). Replication of Human Noroviruses in Stem Cell–Derived Human Enteroids. Science.

[28] Liu D., Deng J., Joshi S., Liu P., Zhang C., Yu Y., Zhang R., Fan D., Yang H., D’Souza D.H. (2018). Monomeric Catechin and Dimeric Procyanidin B2 against Human Norovirus Surrogates and Their Physicochemical Interactions. Food Microbiol..

[29] Lanave G., Catella C., Catalano A., Lucente M.S., Pellegrini F., Fracchiolla G., Diakoudi G., Palmisani J., Trombetta C.M., Martella V. (2024). Assessing the Virucidal Activity of Essential Oils against Feline Calicivirus, a Non-Enveloped Virus Used as Surrogate of Norovirus. Heliyon.

[30] Gassilloud B., Schwartzbrod L., Gantzer C. (2003). Presence of Viral Genomes in Mineral Water: A Sufficient Condition To Assume Infectious Risk?. Appl. Environ. Microbiol..

[31] Sato S., Hisaie K., Kurokawa S., Suzuki A., Sakon N., Uchida Y., Yuki Y., Kiyono H. (2019). Human Norovirus Propagation in Human Induced Pluripotent Stem Cell–Derived Intestinal Epithelial Cells. Cell. Mol. Gastroenterol. Hepatol..

[32] Costantini V., Morantz E.K., Browne H., Ettayebi K., Zeng X.-L., Atmar R.L., Estes M.K., Vinjé J. (2018). Human Norovirus Replication in Human Intestinal Enteroids as Model to Evaluate Virus Inactivation. Emerg. Infect. Dis..

[33] Sato S., Matsumoto N., Hisaie K., Uematsu S. (2020). Alcohol Abrogates Human Norovirus Infectivity in a pH-Dependent Manner. Sci. Rep..

[34] Escudero-Abarca B.I., Goulter R.M., Bradshaw J., Faircloth J., Leslie R.A., Manuel C.S., Arbogast J.W., Jaykus L.-A. (2022). Efficacy of an Alcohol-Based Surface Disinfectant Formulation against Human Norovirus. J. Appl. Microbiol..

[35] Ettayebi K., Salmen W., Imai K., Hagi A., Neill F.H., Atmar R.L., Prasad B.V.V., Estes M.K. (2022). Antiviral Activity of Olanexidine-Containing Hand Rub against Human Noroviruses. mBio.

[36] Kärber G. (1931). Beitrag zur kollektiven Behandlung pharmakologischer Reihenversuche. Arch. Exp. Pathol. Pharm..

[37] Kageyama T., Kojima S., Shinohara M., Uchida K., Fukushi S., Hoshino F.B., Takeda N., Katayama K. (2003). Broadly Reactive and Highly Sensitive Assay for Norwalk-Like Viruses Based on Real-Time Quantitative Reverse Transcription-PCR. J. Clin. Microbiol..

[38] Sauerbrei A. (2020). Bactericidal and Virucidal Activity of Ethanol and Povidone-iodine. MicrobiologyOpen.

[39] Stuart A.D., Brown T.D.K. (2006). Entry of Feline Calicivirus Is Dependent on Clathrin-Mediated Endocytosis and Acidification in Endosomes. J. Virol..

[40] Abou-Hamad N., Estienney M., Chassagnon R., Bon M., Daval-Frerot P., De Rougemont A., Guyot S., Bouyer F., Belliot G. (2023). Biological and Physico-Chemical Characterization of Human Norovirus-like Particles under Various Environmental Conditions. Colloids Surf. B Biointerfaces.

[41] Lewis M.A., Cortés-Penfield N.W., Ettayebi K., Patil K., Kaur G., Neill F.H., Atmar R.L., Ramani S., Estes M.K. (2023). Standardization of an Antiviral Pipeline for Human Norovirus in Human Intestinal Enteroids Demonstrates Nitazoxanide Has No to Weak Antiviral Activity. Antimicrob. Agents Chemother..

