# Supplementary material for: In Vitro Differential Virucidal Efficacy of Alcohol-Based Disinfectants Against Human Norovirus and Its Surrogates
Source: Microorganisms. 2025 Feb 8;13(2):368. doi: 10.3390/microorganisms13020368 (PMC11858694; doi:10.3390/microorganisms13020368)
Supplement: Supplementary file 1 [file microorganisms-13-00368-s001.zip › microorganisms-3397834-supplementary.pdf]

Supplementary Materials

In Vitro Differential Virucidal Efficacy of Alcohol-Based Disinfectants Against Human Norovirus and Its Surrogates

Eri Hiraishi [1,2], Keita Ozaki [1], Moe Yamakami [1], Tempei Akasaka [1] and Hirokazu Kimura [2,*]

[1] Department of Research and Development, Niitaka Co., Ltd, Osaka 532-8560, Japan
[2] Department of Health Science, Gunma Paz University Graduate School of Health Sciences, Takasaki 370-0006, Japan

* Correspondence: h-kimura@paz.ac.jp

1. Evaluation of neutralizing effect of 70 wt% ethanol and alcohol-based disinfectant products on murine norovirus (MNoV) and feline calicivirus (FCV)

A mixture of 10% beef extract and MNoV suspension was prepared in a ratio of 1:1. Five microliters of MNoV suspension containing 5% beef extract was mixed 45 μL of Dulbecco's modified Eagle's medium (DMEM) containing 10% fetal bovine serum (FBS). Forty-five microliters of each disinfectant solution was mixed with 5 μL of DMEM containing 10% FBS. Ten microliters of MNoV mixture and 10 μL of each disinfectant suspension mixture were added to 1,980 μL of DMEM containing 10% FBS. The mixture sample was added to RAW264.7 cells (initial cell seeding concentration: 2.5×10^4 cells/well) cultured for 3 h after seeding on a 96-well microtiter plate. The cells were cultured at 37°C under an atmosphere containing 5% CO_2 for 4 days. Cytopathic effect was observed, and median tissue culture infectious dose ($TCID_{50}$) was calculated from cytopathic effect using the Behrens–Kärber method.

For FCV, a similar experiment was performed by replacing MNoV with FCV. We used a modified of Eagle's minimum essential medium (Opti-MEM) instead of DMEM containing 10% FBS. The mixture sample with the disinfectant, virus suspension, and medium was added to confluent monolayers of Crandell–Rees feline kidney cells (initial cell seeding concentration: 8.0×10^3 cells/well) cultured at 37°C under 5% CO_2 atmosphere for 3–4 day after seeding on a 96-well microtiter plate.

Table S1. Evaluation of the neutralizing efficiency of 70 wt% ethanol and disinfectant products against MNoV and FCV.

**Disinfectant solutions**	**Viral infectivity titer of MNoV [log_{10} $TCID_{50}$/50 μL] [a)]**		**Viral infectivity titer of FCV [log_{10} $TCID_{50}$/100 μL] [a)]**	
	**Untreated**	**Neutralization**	**Untreated**	**Neutralization**
70 wt% ethanol	7.3 (0.1)	7.3 (0.1)	6.6 (0.1)	6.5 (0.5)
Product A	6.3 (0.2)	6.1 (0.1)	6.5 (0.2)	6.5 (0.3)
Product B	7.1 (0.1)	7.4 (0.4)	6.5 (0.2)	6.3 (0.3)
Product C	6.5 (0.5)	6.3 (0.4)	6.4 (0.2)	6.4 (0.1)
Product D	7.1 (0.1)	6.9 (0.1)	6.4 (0.3)	6.2 (0.3)

a) Data are shown as mean, and standard deviations are shown in parentheses.

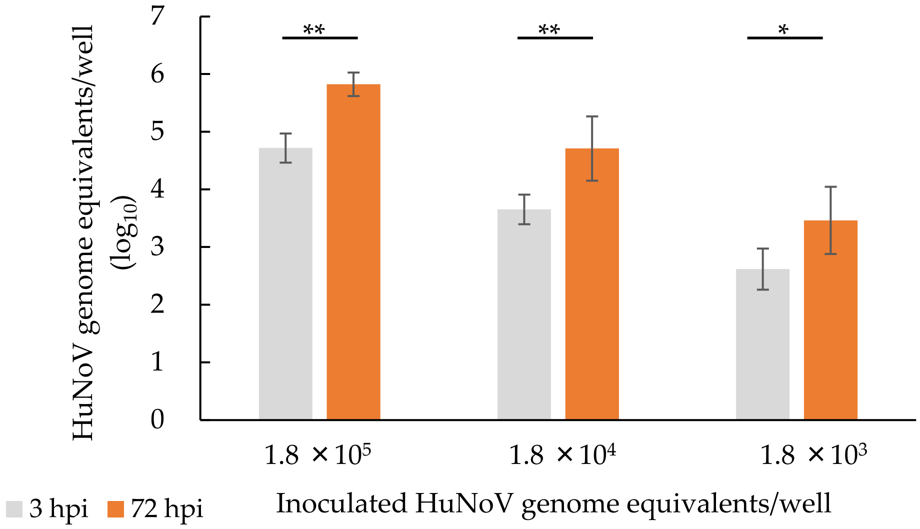

Figure S1. Correlation between the amount of human norovirus (HuNoV) RNA in the HuNoV infection experiments and HuNoV RNA proliferation in monolayer intestinal epithelial cells. We used the indicated amounts of GII.17 HuNoV RNA (1.8×10^5, 1.8×10^4, and 1.8×10^3 GEs/well) for HuNoV infection experiments. In total, 10 μL of diluted HuNoV suspension was mixed with 1,000 μL of Advanced DMEM/F-12 and 90 μL of PBS. Subsequent operations were performed in the same manner as reported in Materials and Methods. Viral RNA was extracted from both cells and supernatants at 3 and 72 hpi. Viral replication was determined by quantifying RNA levels using reverse transcriptase quantitative PCR (RT-qPCR). Values on the vertical axis indicate the amount of HuNoV RNA at 3 and 72 hpi. Values on the horizontal axis indicate the initial inoculated amount of HuNoV RNA into monolayer of F-hiSIECs. Results are presented as the mean ± standard deviation. Each experiment was performed two times. The compiled data represent the RNA mean of three wells. Data were compared using the Welch's *t* test. Asterisks indicate significant differences between 3 and 72 hpi (* $p < 0.05$ and ** $p < 0.01$).

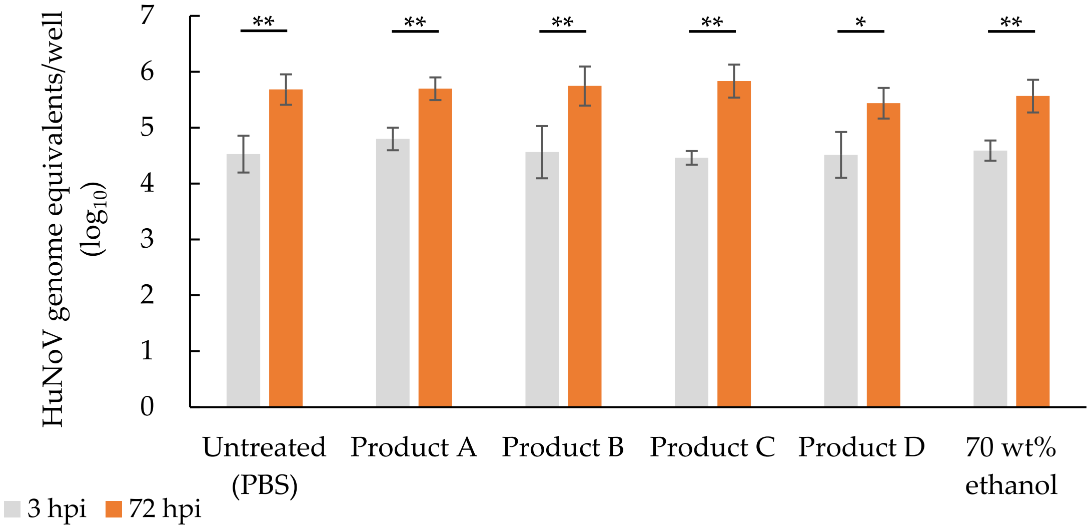

Figure S2. Evaluation of the neutralizing efficiency of virucidal activity of products and 70 wt% ethanol against GII.17 HuNoV. Briefly, 10 μL of HuNoV suspension containing 3.6 × 10^4 GEs/μL was added to a mixture of 90 μL of each disinfectant solution and 1,000 μL Advanced DMEM/F-12. The dilution was ultracentrifuged using Optima MAX-TL with a type TLA-55 rotor at 55,000 rpm for 90 min at 4°C. The supernatant was removed, and the pellet was resuspended in 100 μL of culture medium for F-hiSIECs containing 500 μM sodium glycochenodeoxycholate. One-half of the resuspended mixture (50 μL) was transferred to two confluent monolayers of F-hiSIECs seeded on a 96-well microtiter plate. Subsequent sampling and quantification of the HuNoV genome was performed as described in the Materials and Methods. Values on the vertical axis indicate the amount of HuNoV RNA at 3 and 72 hpi. Labels on the horizontal axis indicate the disinfectant solutions used in the HuNoV inactivation experiments. The amount of HuNoV RNA at 72 hpi proliferated to 13-, 8-, 12-, 28-, 7-, and 11-fold compared with those at 3 hpi by PBS, product A, product B, product C, product D, and 70 wt% ethanol treatment, respectively. Results are presented as the mean ± standard the deviation. Each experiment was performed two times. The compiled data represents the RNA mean of three wells. Data were compared using Welch's *t* test. Asterisks indicate significant differences between 3 and 72 hpi (* $p < 0.05$ and ** $p < 0.01$).
